# Computational Modeling of the Metabolic States Regulated by the Kinase Akt

**DOI:** 10.3389/fphys.2012.00418

**Published:** 2012-11-21

**Authors:** Ettore Mosca, Roberta Alfieri, Carlo Maj, Annamaria Bevilacqua, Gianfranco Canti, Luciano Milanesi

**Affiliations:** ^1^Institute for Biomedical Technologies, Consiglio Nazionale delle Ricerche SegrateMilano, Italy; ^2^Department of Informatics, Systems and Communication, Università di Milano-BicoccaMilano, Italy; ^3^Università Telematica San Raffaele RomaMilano, Italy; ^4^Department of Medical Biotechnology and Translational Medicine, Università degli Studi di MilanoMilano, Italy

**Keywords:** PI3K/Akt/mTOR pathway, metabolism, kinetic models, glycolysis, cancer

## Abstract

Signal transduction and gene regulation determine a major reorganization of metabolic activities in order to support cell proliferation. Protein Kinase B (PKB), also known as Akt, participates in the PI3K/Akt/mTOR pathway, a master regulator of aerobic glycolysis and cellular biosynthesis, two activities shown by both normal and cancer proliferating cells. Not surprisingly considering its relevance for cellular metabolism, Akt/PKB is often found hyperactive in cancer cells. In the last decade, many efforts have been made to improve the understanding of the control of glucose metabolism and the identification of a therapeutic window between proliferating cancer cells and proliferating normal cells. In this context, we have modeled the link between the PI3K/Akt/mTOR pathway, glycolysis, lactic acid production, and nucleotide biosynthesis. We used a computational model to compare two metabolic states generated by two different levels of signaling through the PI3K/Akt/mTOR pathway: one of the two states represents the metabolism of a growing cancer cell characterized by aerobic glycolysis and cellular biosynthesis, while the other state represents the same metabolic network with a reduced glycolytic rate and a higher mitochondrial pyruvate metabolism. Biochemical reactions that link glycolysis and pentose phosphate pathway revealed their importance for controlling the dynamics of cancer glucose metabolism.

## Introduction

Growth factors and nutrients are required for cell growth and proliferation in multicellular organisms. As a consequence of growth factors withdrawal normal cells undergo apoptosis, while most transformed cells escape the regulatory mechanisms and acquire the ability to proliferate even in the absence of growth signals (Edinger, 2005). In both normal and cancer cells the onset of proliferation induces important changes in cellular metabolism. Therefore metabolic activities in proliferating cells are fundamentally different from those in non-proliferating cells (DeBerardinis et al., 2008; Lunt and Vander Heiden, 2011).

The correlation between signal transduction pathways and cellular metabolism is mediated by some key components of the growth factor-induced cascades; typically these elements are protein kinases at the core of physiology and disease. Several growth factor-induced signal transduction pathways have been characterized so far and, in particular, the phosphoinositide 3-kinase (PI3K) is a key component downstream of the receptor tyrosine kinases (RTKs; Cantley, 2002). The PI3K is responsible for the production of 3-phosphoinositide lipid second messengers including phosphoinositol trisphosphate (PIP3) at the cell membrane. PIP3, in turn, contributes to the recruitment and activation of a wide range of downstream targets, among which the serine-threonine protein kinase Akt, also known as protein kinase B (PKB; Nicholson and Anderson, 2002; Gonzalez and McGraw, 2009). Akt/PKB is phosphorylated at two sites, one within the T-loop of the catalytic domain by the phosphoinositide-dependent kinase 1 (PDK1) and the other within the carboxyl terminal hydrophobic domain by the mammalian target of rapamycin complex 2 (mTORC2; Alessi et al., 1997; Sarbassov et al., 2005).

**Figure 1:**
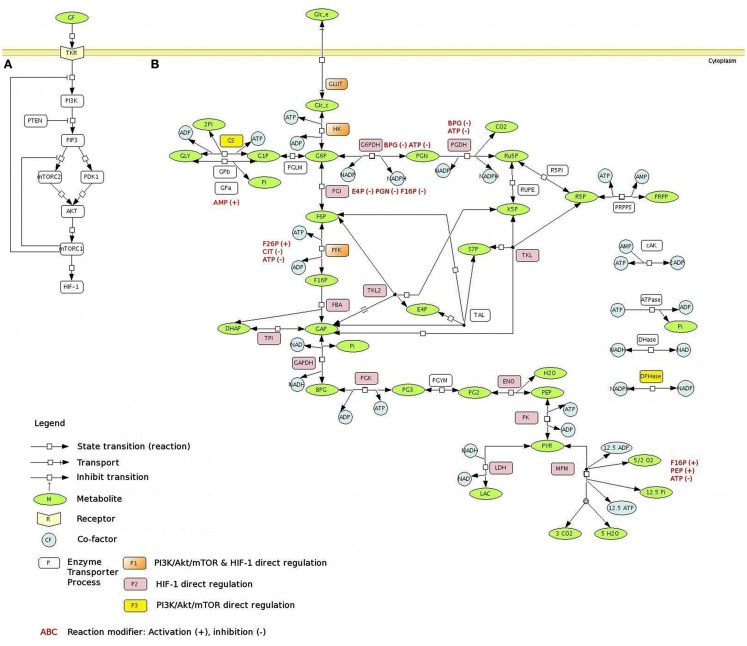
**The PI3K/Akt/mTOR pathway regulates central carbon metabolism**. **(A)** PI3K/Akt/mTOR pathway. Signaling through the PI3K/Akt/mTOR pathway begins with the activation of RTKs in response to growth factors, leading to auto-phosphorylation on tyrosine residues and trans-phosphorylation of adaptor proteins. The PI3K is responsible for the production of 3-phosphoinositide lipid second messengers, including PIP3, which contributes to the activation of many downstream targets, such as PDK1 and mTORC2. Both PDK1 and mTORC2 activate, through phosphorylation in different sites, the serine-threonine protein kinase Akt. Akt regulates multiple functions including cellular metabolism, by promoting cell growth and proliferation through the activation of mTORC1, which also enhances the transcriptional activity of HIF-1α. Dashed lines represent the negative regulation of the PI3K/Akt/mTOR pathway by the action of mTORC1 feedback mechanism. **(B)** The metabolic network with the main reactions of glucose metabolism. A schematic representation of the glucose metabolism network, using the SBGN notation (LeNovere:2009]), is presented. The main pathways involved in the glucose metabolism are considered: glycolysis, PPP, the glycogen synthesis and degradation, lactate, and MPM branches. The metabolic targets regulated by PI3K/Akt/mTOR pathway are represented on the network: the PI3K/Akt/mTOR direct regulation is presented in yellow; the PI3K/Akt/mTOR indirect regulation (via HIF-1α) is presented in pink; the PI3K/Akt/mTOR direct and indirect regulation is presented in orange. All the PI3K/Akt/mTOR direct and indirect targets considered here are positively regulated, with the only exception of the MPM. Allosteric regulators (modifiers), activators (+), or inhibitors (−), are depicted in red. Metabolites – ADP, adenosine diphosphate; AMP, Adenosine Monophosphate; ATP, Adenosine Triphosphate; BPG, 1,3-bisphosphoglycerate; DHAP, dihydroxyacetone phosphate; E4P, erythrose-4-phosphate; F6P, fructose-6-phosphate; F16P, fructose-1,6-bisphosphate; G1P, glucose-1-phosphate; G6P, glucose-6-phosphate; GAP, glyceraldehyde-3-phosphate; GLC_c, cytoplasmic glucose; GLC_e, extracellular glucose; GLY, glycogen; LAC, lactate; NAD, nicotinamide adenine dinucleotides; NADH, nicotinamide adenine dinucleotides; NADP, nicotinamide adenine dinucleotide phosphate; NADPH, nicotinamide adenine dinucleotide phosphate; Pi, inorganic phosphate; PEP, phosphoenolpyruvate; PG2, 2-phosphoglycerate; PG3, 3-phosphoglycerate; PGN, 6-phosphogluconolactone; PRPP, phosphoribosylpyrophosphate; PYR, pyruvate; R5P, Ribose-5-phosphate; RU5P, ribulose-5-phosphate; X5P, xylulose-5-phosphate; S7P, sedoheptulose-7-phosphate; reactions – AK, adenylate Kinase; ATPase, ATP hydrolysis; DHase, NADH oxidation; DPHase, NADPH oxidation; ENO, Enolase; G6PDH, glucose-6-P dehydrogenase; GAPDH, glyceraldehyde dehydrogenase; GLUT, glucose transporter; GPa, glycogen phosphorylase A; GPb, glycogen phosphorylase b; GS, Glycogen synthase; FBA, fructose-6-P aldolase; HK, Hexokinase; LDH, lactate dehydrogenase; MPM, mitocondrial pyruvate metabolism; PFK, phosphofructo-kinase; PGDH, phoshogluconolactone dehydrogenase; PGI, phosphoglucoisomerase; PGK, phosphoglycerate kinase; PGLM, phosphoglucomutase; PGYM, 3-phosphoglycerate mutase; PK, pyruvate kinase; PRPPS, phosphoribosylpyrophosphate synthetase; R5PI, ribose-5-P isomerase; RUPE, ribulose-phosphate-3 epimerase; TAL, transaldolase; TKL, transketolase, reaction I; TKL2, transketolase, reaction II; TPI, triose-phosphate isomerase.

Fully activated Akt/PKB translocates from the cell membrane to the cytosol and nucleus where it phosphorylates its substrates (Manning and Cantley, 2007) to regulate multiple functions including cellular metabolism (Figure 1A). One of the chief mechanisms of Akt/PKB promoting cell growth and proliferation is through the activation of mTOR complex 1 (mTORC1), which is regulated by both nutrients and growth factor signaling (Wullschleger et al., 2006; Zoncu et al., 2011). Moreover, mTORC1 directly enhances the transcriptional activity of hypoxia-inducible factor 1α (HIF-1α; Land and Tee, 2007). HIF-1α is known to control the expression of several genes involved in energy metabolism, apoptosis, angiogenesis, and metastasis (Carmeliet et al., 1998; Pugh and Ratcliffe, 2003; Marín-Hernández et al., 2009).

Negative regulation of the PI3K/Akt/PKB pathway is primarily accomplished through the action of the PTEN tumor suppressor protein, a lipid and protein phosphatase whose main lipid substrate is PIP3 (Song et al., 2012). Recently, a crucial mTORC1-dependent feedback mechanism has been elucidated (Howell and Manning, 2011). According to the current knowledge mTORC1 can exert a negative regulation of its upstream signaling molecules as depicted in Figure 1A.

Overall, the role of the PI3K/Akt/PKB signaling pathway in oncogenesis has been extensively investigated and altered expression or mutations of many components of this pathway have been implicated in human cancer (Vivanco and Sawyers, 2002; Carnero, 2010). Indeed, expression of constitutively active forms of Akt/PKB can prevent cell death upon growth factor withdrawal. PI3K/Akt/mTOR-mediated survival relies on a profound metabolic adaptation, including aerobic glycolysis (known as Warburg effect).

In the last decade, many efforts have been made to improve the understanding of the control of glucose metabolism. High-rate glycolysis is supported by a number of molecular alterations, which are either unique to cancer in an otherwise healthy organism or druggable with controllable toxicities (Porporato et al., 2011). In general, the goal is to find a therapeutic window between proliferating cancer cells and proliferating normal cells (Vander Heiden, 2011) in the development of successful cancer therapies targeting cellular metabolism. To be an attractive candidate for cancer therapy, there must be a significant difference in enzyme activity between cancer cells and normal proliferating cells (Hamanaka and Chandel, 2012).

In this context, we have modeled the link between the PI3K/Akt/mTOR pathway and metabolism, specifically the glycolytic rate, lactic acid production, and nucleotide biosynthesis. Computational models are a tool to understand, control, and predict the complex behavior of biological systems; for example, computer-based simulations can be a powerful alternative to wet experiments for exploring the impact of perturbations, such as drug treatments, on a molecular network (Kreeger and Lauffenburger, 2010). Indeed, dynamical modeling of complex biological systems has been widely used in the last decade. In particular, Ordinary Differential Equations (ODEs) have been exploited as a standard numerical method in many successful examples (Novak and Tyson, 1993; Tyson et al., 2001; Bazil et al., 2010). Before becoming a predictive tool, a model must be defined and corroborated considering the available knowledge and experimental data. However, current experimental techniques limit our ability to obtain *in vivo* information about some aspects of metabolic pathways. In fact, while metabolic fluxes can be measured *in vivo* using metabolically active molecules labeled with stable isotopes, it is hard or even impossible to obtain accurate *in vivo* measures of enzyme properties. Therefore no existing experimental dataset contains all the information for the biochemical processes and molecular species under analysis. Nevertheless, several experimental datasets, collected using a variety of tissue sources, techniques and experimental conditions (e.g., *in vitro*), are available for metabolic pathways. Therefore, these data can be carefully integrated to fill the lack of information, so that models can be optimized by means of various simulation and analysis tools (Ghosh et al., 2011). With the addition of new experimental datasets, the model is refined through possible modifications of its structure or parameter values.

Several models have been developed to describe the glucose metabolism (Mosca et al., 2012). To study the accelerated glycolysis of human cancer cells, Marín-Hernández et al. (2011) constructed a model supported by experimental data for enzyme kinetics, steady state pathway metabolite concentrations, and metabolic fluxes. This model was used as the base model for our work due to the type of cells used (a human cancer cell line) and the amount of experimental data collected in the same condition. To accomplish the task of modeling the metabolic effects of the PI3K/Akt/mTOR pathway, we extended the network configuration and replaced simplified rate equations for glycolytic branches with thermodynamically consistent and detailed representations. Subsequently, some parameter values were optimized to obtain a quantitative reproduction of the metabolic state (fluxes and metabolites) of a proliferating cancer cell line. In the resulting extended model, we modulated the activity of PI3K/Akt/mTOR in order to simulate the effects of the kinase levels over the rates of the processes controlled by its (direct and indirect) targets. Then, we identified the reactions exerting a major control over the metabolic network at two different rates of glucose metabolism, corresponding to high and low PI3K/Akt/mTOR activity. Enzymes that have a different relevance in the two metabolic states represent potential targets for a more selective control of the system.

## Results

### Model development

The model represents the dynamics of the metabolic network composed by the main metabolites and biochemical processes (transports and reactions) of the glucose metabolism: the glucose transport, the glycolytic reactions, the lactic acid production, the glycogen synthesis/degradation, and the pentose phosphate pathway (PPP; Figure 1B). The rates of the biochemical processes considered in the model are defined by taking into account the details of the respective kinetic mechanisms. Therefore, the main activators and inhibitors forming feedback and feedforward regulations are also considered. To maintain thermodynamic consistency and since equation simplification by irreversibility limits the possible steady states allowed in the system, biochemical processes are represented as thermodynamically balanced and reversible. This approach was also important to enable the reproduction of the wide range of glycolytic rates observed in relation to the regulatory activities operated by oncogenes (Levine and Puzio-Kuter, 2010). The only exception is the ATPase reaction, which is used here as elsewhere (e.g., Lambeth and Kushmerick, 2002; Bazil et al., 2010; Marín-Hernández et al., 2011) to simulate, with a unique flux, all the ATP consuming reactions that are not detailed in the model.

The model is a 28 state system of differential algebraic equations (DAEs) that consists in 25 ODEs and 3 algebraic equations to calculate ADP, NADH, and NADPH concentrations (see Appendix). As previously mentioned, this model is primarily based on a recent cancer glycolysis model of human cervical cancer HeLa cells (Marín-Hernández et al., 2011). The model of HeLa cells glycolysis was developed to reproduce a specific metabolic steady state, making use of detailed rate equations for glycolysis and simplified irreversible reactions for the PPP, the glycogen synthesis and degradation, and the mitochondrial pyruvate metabolism (MPM) branches. Since these glycolytic branches also play an important role in relation to the metabolic states determined by PI3K/Akt/mTOR signaling, we extended the network of reactions substituting simplified branches with thermodynamically balanced and reversible kinetics. In particular, rate equations for PPP, glycogen synthesis, and degradation were defined as in the model of Holzhütter (2004); the kinetics of phosphoglucomutase (PGLM) and glycogen synthase (GS) were derived from Li et al. (2010b); the glycogen phosphorylase (GP) kinetics from Lambeth and Kushmerick (2002); the MPM was defined using a common modular rate law (Liebermeister et al., 2010). The model is available in BioModels database (Li et al., 2010a) with identifier MODEL1210150000.

### Model fitting

The model was optimized assembling a dataset that represents the typical traits of a cancer cell growing in condition of non-limiting glucose: increased glycolytic rate, active synthesis of NADPH and nucleotides, predominant metabolization of glucose to lactate rather than to CO_2_ and H_2_O through the tricarboxylic acid cycle (Levine and Puzio-Kuter, 2010).

As in several other recent computational models of metabolic pathways (e.g., Bazil et al., 2010; Li et al., 2010b), we adjusted only a small subset of the parameters appearing in the model equations and, more precisely, we focused on the maximum rates of forward reactions (Table 1). Biologically, this can be interpreted as the correction of enzyme concentrations or enzyme activities in order to explain, with the equations used in the model, the metabolic values (fluxes and concentrations) measured experimentally. Crucially, by constructing the model taking into account the Haldane constraint, which relates the equilibrium constant of a biochemical reaction with (forward and reverse) maximum rates and reactant constants (Appendix), the adjustment/optimization of the parameter values will not violate the thermodynamics of any reaction or of the entire network (Lambeth and Kushmerick, 2002), but only physiologically appropriate kinetic parameter values will result in reasonable fluxes and accurate dynamics.

**Table 1:** **Maximum reaction rates in conditions H and L**.

Reaction	Definition	Condition H		Condition L	
		*V*_f_ (nmol/min/mg)	*s*	*V*_f_ (nmol/min/mg)	*s*
AK	Adenylate kinase	1.412e+02	3.449e−02	1.412e+02	3.449e−02
ATPase^a^	ATP hydrolysis	6.210e+03	2.292e−01	6.210e+03	2.373e−01
DHase	NADH oxidation	4.982e+06	1.171e−03	4.982e+06	2.278e−03
DPHase	NADPH oxidation	1.278e+05	6.963e−02	7.413e+04	3.430e−02
ENO	Enolase	1.609e+02	3.627e−04	9.330e+01	4.074e−04
FBA	Fructose-6-P aldolase	1.463e+01	6.639e−04	8.484e+00	1.491e−03
G6PDH	Glucose-6-P dehydrogenase	1.008e+00	1.181e+00	5.846e−01	7.600e−01
GAPDH	Glyceraldehyde dehydrogenase	1.091e+02	2.345e−02	6.329e+01	2.696e−02
GLUT	Glucose transporter	2.303e+01	4.271e−01	1.336e+01	5.256e−01
GPa	Glycogen phosphorylase a	3.347e−02	3.602e−02	3.347e−02	3.790e−02
GPb	Glycogen phosphorylase b	1.049e−02	3.679e−02	1.049e−02	3.962e−02
GS	Glycogen synthase	3.204e+04	3.934e−02	1.858e+04	6.016e−02
HK	Hexokinase	8.685e+01	7.173e−02	5.037e+01	5.998e−02
LDH	Lactate dehydrogenase	3.403e+02	3.331e−03	1.974e+02	3.647e−03
MPM	Mitochondrial pyruvate metabolism	9.801e+06	1.142e−01	1.137e+07	1.423e−01
PFK	Phosphofructo-kinase	1.076e+02	5.706e−02	6.243e+01	1.138e−01
PGDH	Phoshogluconolactone dehydrogenase	3.102e+01	5.136e−03	1.799e+01	6.160e−04
PGI	Phosphoglucoisomerase	7.778e+03	3.709e−02	4.511e+03	3.216e−03
PGK	Phosphoglycerate kinase	7.341e+01	8.065e−03	4.258e+01	9.261e−03
PGLM	Phosphoglucomutase	7.364e+00	3.351e−02	7.364e+00	1.651e−02
PGYM	Phosphoglycerate mutase	1.540e+02	2.232e−03	1.540e+02	2.489e−03
PK	Pyruvate kinase	2.781e+01	1.070e−07	1.613e+01	4.182e−07
PRPPS	Phosphoribosylpyrophosphate synthetase	5.104e+01	5.898e−01	5.104e+01	5.439e−01
R5PI	Ribose-5-P isomerase	7.646e+01	2.643e−02	7.646e+01	6.134e−02
RUPE	Ribulose-phosphate epimerase	1.471e+00	1.156e−03	1.471e+01	2.467e−03
TAL	Transaldolase	5.827e+01	7.061e−04	5.827e+01	1.040e−04
TKL	Transketolase (reaction I)	1.056e+03	5.345e−04	6.124e+02	1.180e−03
TKL2	Transketolase (reaction II)	1.761e+01	4.674e−01	1.021e+01	2.420e−02
TPI	Triose-phosphate isomerase	5.976e+00	2.779e−04	3.466e+00	6.055e−04

*Maximum rates of the forward reactions (*V*_f_) and mean first-order local sensitivities (*s*) of steady state fluxes in relation to perturbation of *V*_f_ values. ^a^min^−1^mg^−1^*.

**Figure 2:**
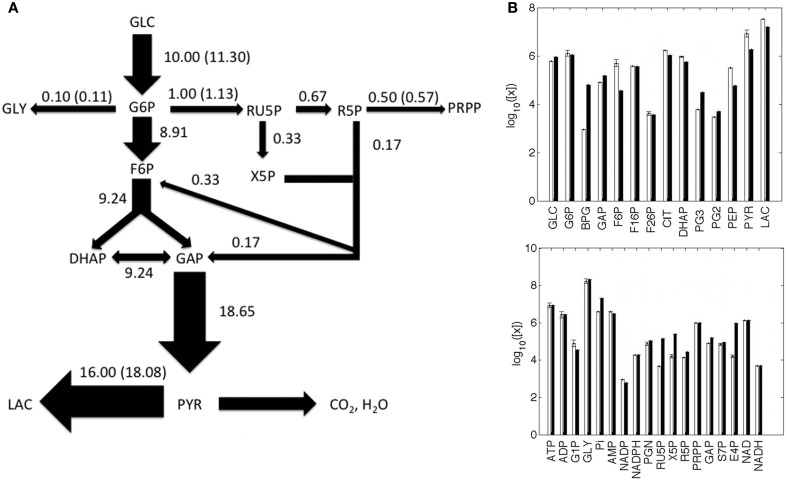
**Metabolic fluxes and metabolite concentrations**. **(A)** A Simplified illustration of the metabolic network, where the width of the arrows is proportional to the predicted flux values, which are reported close to the respective arrows; experimental values are shown in parentheses; fluxes are in nmol/min/mg unit. **(B)** Predicted (white) and experimental (black) metabolite concentrations, reported as the log_10_ of nM values. The full list of predicted and experimental fluxes and concentration values in conditions H and L is available in the Appendix.

Metabolite concentrations and metabolic fluxes were primarily collected from two experimental studies on human cervix cancer HeLa cells (Reitzer et al., 1980; Marín-Hernández et al., 2011). Reitzer et al. (1980) measured the flux of sugar carbon per unit of protein synthesized in the major pathways of metabolism of HeLa cells growing on 10 mM glucose. Under this condition, they observed that the 10% of the glucose converted to glucose-6-P, which was quantified as 11.3 nmol/min/mg, is diverted toward the oxidative arm of the pentose cycle, 5% to purine metabolism and nucleotide biosynthesis, 1% to glycogen, and 80% to lactate (Figure 2A). The optimized model provided a satisfactory representation of the experimental data considered, in terms of fluxes (Figure 2A) and metabolite concentrations (Figure 2B).

### *In silico* modulation of PI3K/Akt/mTOR activity

We considered the most established metabolic targets regulated by PI3K/Akt/mTOR and by HIF-1α, as indicated in Figure 1B. The PI3K/Akt/mTOR pathway activation increases the synthesis of GLUT, the main glucose transporter in most cell types (Kohn et al., 1996), and enhances its transcription and its translocation from the cytosol to the plasma membrane, increasing glucose uptake (Barthel et al., 1999; Rathmell et al., 2003). Upon PI3K/Akt/mTOR stimulation, the activity of the hexokinase (HK) is enhanced (Elstrom et al., 2004). PI3K/Akt/mTOR has also effects on other steps of glycolysis; in fact, it has been shown that increasing GLUT and HK expression does not enhance the glycolytic flux to the observed levels with constitutive activation of PI3K/Akt/mTOR (Rathmell et al., 2003). Glycolysis downstream targets of PI3K/Akt/mTOR include PFK2; phosphorylation and activation of PFK2 lead to allosteric activation of PFK1 (Deprez et al., 1997). Moreover, PI3K/Akt/mTOR inhibits GSK, the GS kinase-3beta (Yoeli-Lerner et al., 2009), which inhibits the GS; as a consequence, PI3K/Akt/mTOR has indirect positive effects on the glycogen synthesis. Also the ATP-citrate lyase, the primary enzyme responsible for the synthesis of cytosolic acetyl-CoA, is a substrate for PI3K/Akt/mTOR (Berwick et al., 2002). An important target of PI3K/Akt/mTOR is HIF-1α, which can be overexpressed also under non-hypoxic conditions through PI3K/Akt/mTOR (Lee et al., 2008); in turn, HIF-1α is responsible for the positive regulation of several enzymes of the central metabolism: almost all the glycolytic enzymes (Semenza et al., 1994), G6PDH, PGDH (Guo et al., 2009), TKL (Zhao et al., 2010), and LDH (Firth et al., 1995); HIF-1α also stimulates the inhibition of PDH (Biswas et al., 2010).

To model the metabolic effects of the PI3K/Akt/mTOR signaling pathway, we modified the rate equations for the biochemical processes regulated by the targets of PI3K/Akt/mTOR. Rate equations were modified on the basis of the specific effect PI3K/Akt/mTOR exerts over each of its targets. If PI3K/Akt/mTOR positively (negatively) regulates the protein concentration, we increased (decreased) the value of the maximum rate of the forward reaction (*V*_f_) of the corresponding process, because this quantity is directly proportional to the total protein concentration (Sauro, 2011). This is the case of GLUT, GS, glycolytic enzymes, and PPP enzymes (Figure 2). The enhancement of PFK activity mediated by PI3K/Akt/mTOR is also exerted through F26P, one of PFK allosteric activators, and therefore we increased the F26P concentration. The positive relation between PI3K/Akt/mTOR and fatty acid synthesis was taken into account increasing the rate of NADPH consuming reactions (fatty acid synthesis requires NADPH), while the inhibition of PDH due to PI3K/Akt/mTOR was simulated reducing the rate of the MPM process.

**Figure 3:**
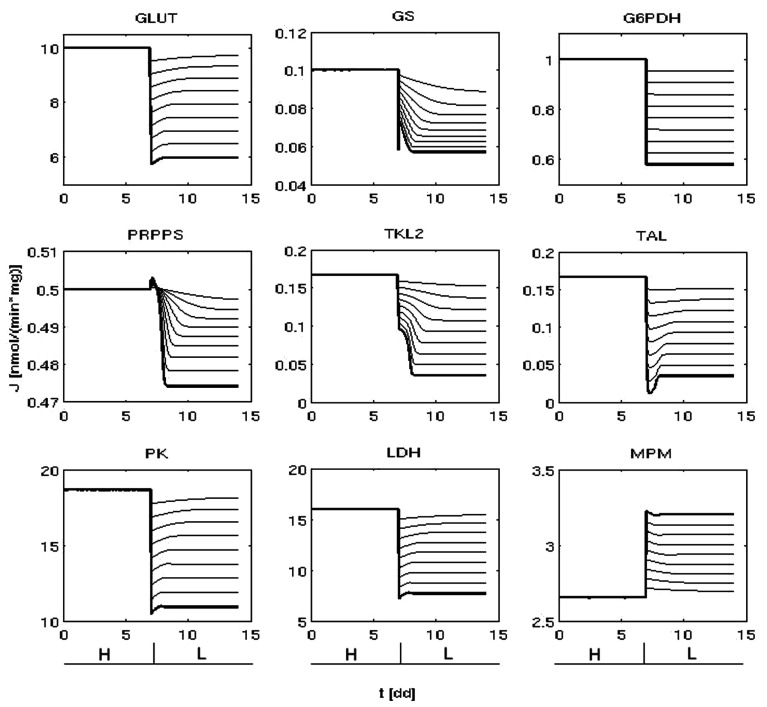
**Lower metabolic fluxes are obtained reducing PI3K/Akt/mTORs activity**. Time variation of some representative fluxes (nmol/min/mg) before (condition H) and after the reduction of PI3K/Akt/mTOR signaling (at *t* ( 7dd). The thick lines indicate the dynamics that lead to the representative condition L (right).

Starting from the metabolic state representing the typical traits of a cancer cell growing in condition of non-limiting glucose (Figure 2) and modulating PI3K/Akt/mTOR activity, we obtained several metabolic steady states (Figure 3). Coherently with what is observed experimentally (DeBerardinis et al., 2008), the flux through glycolysis, glycogen synthesis, and the portion of pyruvate metabolized toward lactate increased as the PI3K/Akt/mTOR activity was increased and *vice versa*. We considered two metabolic steady states as representatives of two different glycolytic rates, corresponding to high or low PI3K/Akt/mTOR activity: the first, representing a cancer cell line where PI3K/Akt/mTOR promotes a high-rate of glucose metabolism (Figures 2 and 3, condition H); the second characterized by a lower glycolytic rate due to a reduced PI3K/Akt/mTOR signal (Figure 3, condition L).

In relation to condition H, condition L was selected for its reasonable reproduction of the available experimental data and for conserving the direction of the metabolic fluxes. In fact, on the one hand the variation of glycolysis and lactic acid production rate between state H and L was respectively of ∼1.7- and ∼2.0-fold (Figure 3; Appendix), close to the respective values of ∼1.5 and ∼3.0 recently measured by Fan et al. (2010) after activation of Akt. On the other hand, by conserving the same flux direction between conditions H and L we were able to exclude effects due to a different configuration of the flow of mass within the system, enabling a meaningful comparison focused on metabolic rates between two states that reproduce the metabolic variations measured in relation to PI3K/Akt/mTOR activity (for instance, other possible configurations could be: the non-oxidative arm of the PPP that extracts part of the glycolytic flux and redirects it toward nucleotide biosynthesis; glycogen degradation higher than glycogen synthesis).

### Local sensitivity analysis

A first-order sensitivity analysis (SA) on the model was performed to determine how robust the steady states under analysis were in relation to local perturbations in the parameter values. These sensitivity indexes represent a measure of the degree to which the steady states H and L are sensitive to the value of individual parameters. A high sensitivity value indicates that changing a given parameter determines significant changes to the considered dynamics. The averages (median) of the sensitivity indexes, of all the 29 adjustable parameters, are respectively 3.198e-03 and 7.320e-04 for conditions H and L (Table 1). Therefore, on average a small perturbation of a given parameter determines a change in the steady state fluxes that is approximately three orders of magnitude less than the perturbation.

### Comparison of the enzyme control over the system at different rates of glucose metabolism

To study the global sensitivity of conditions H and L in relation to each biochemical process of the metabolic network (listed in Table 1), we calculated, for each possible pairs of biochemical processes, the Derivative Based Global Sensitivity Measures (DGSMs; Kucherenko et al., 2009) that summarize the (total) effect exerted by a reaction on the steady state flux of another reaction around the metabolic steady states H and L. Conversely form the Metabolic Control Analysis (MCA; Fell, 1997), DGSMs are a method for SA that copes with model non-linearities and interactions between the quantities under analysis. In fact, this method does not require a linearization of the system and considers the effect of perturbing more than one quantity at the same time, but it is computationally more intensive than MCA (see Materials and Methods).

**Figure 4:**
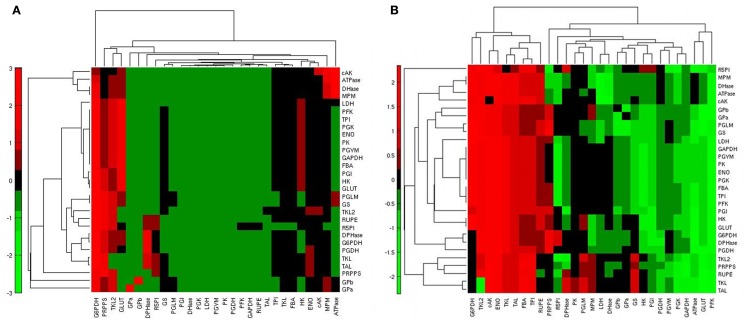
**Heatmap of the steady state flux sensitivity to reactions**. **(A)** Sensitivities of the steady state fluxes (rows) in relation to the reactions (columns) in condition H; red: high sensitivity; green: low sensitivity. **(B)** Differential ranking of steady state flux sensitivities (rows) to reactions (columns) when comparing conditions H and L; red: high sensitivity in condition H; green: high sensitivity in condition L.

In state H, steady fluxes are more sensitive to enzymes of the PPP (Figure 4A): G6PDH, first enzyme of the PPP and also target of p53 (Jiang et al., 2011); PRPPS, which catalyzes the phosphoribosylation of ribose-5-phosphate to 5-phosphoribosyl-1-pyrophosphate, a metabolite that is necessary for purine metabolism and nucleotide biosynthesis; TKL, a thiamine-dependent enzyme that channels sugar phosphates between glycolysis and the PPP. GLUT, the source of mass of the system, also exerts a major control. With the exceptions of HK, FBA, TPI, and ENO, the variation of other glycolytic enzyme activity appears to exert a similar effect on the steady state metabolic fluxes in the two conditions. In condition L, steady state fluxes are still sensitive to GLUT, G6PDH, and PRPPS, even if the amount of control differs; in contrast, TKL loses its role and PFK, HK, and ATPase emerge as important players for system dynamics.

**Figure 5:**
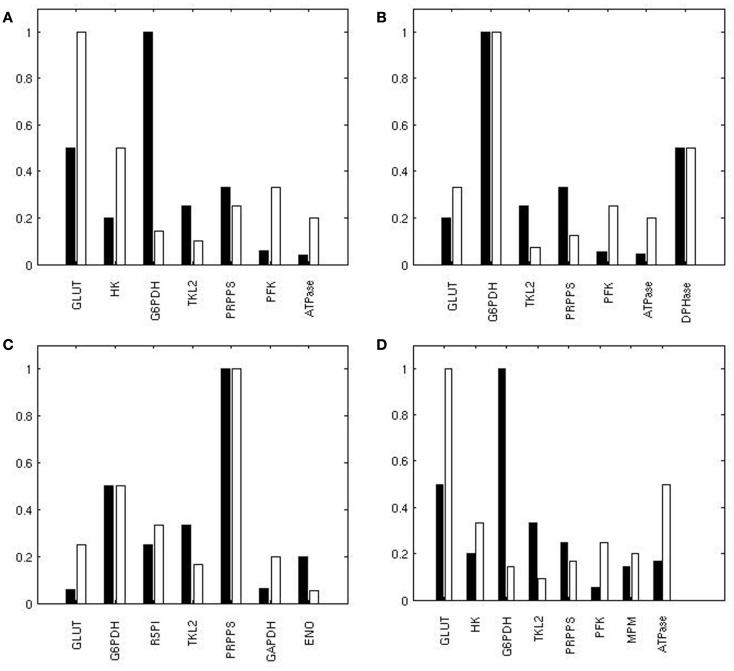
**Reactions exerting the most of the control over the metabolic network**. Ranking of the top reactions having the highest influence on the flux of GLUT **(A)**, G6PDH **(B)**, PRPPS **(C)**, and LDH **(D)** in H (black) and L (white). A value of 1 corresponds to the best rank.

To identify the reactions exerting a different control over the system when using the two conditions H and L, we calculated, for each reaction, the normalized difference in the ranking of all the reactions based on their relevance in state H and L. When comparing the ranking of the sensitivity indexes between the two metabolic steady states, we observed that the PPP enzymes G6PDH and TKL showed a prominent role in condition H; ENO, responsible of the conversion between PG2 and PEP, also had a relatively higher control in H in comparison to L (Figure 4B). Conversely, in condition L the system is more sensitive to GLUT, PFK, and to some of the enzymes of the glycolytic “pay off” phase. This trend is also evident when considering the top ranked enzymes for the control of specific steps (Figure 5).

**Figure 6:**
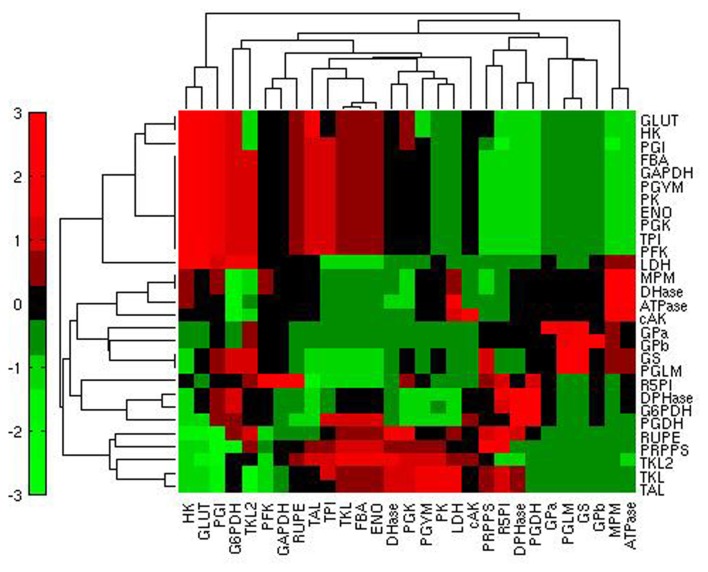
**Heatmap of the relative log sensitivities of steady state fluxes (rows) in relation to reactions (columns) in condition H**. Red: high sensitivity; green: low sensitivity.

Another interesting question that arises with the type of SA we have performed concerns the reactions having a high control over a specific flux or set of fluxes. To this aim we calculated, for each flux, the relative log sensitivity (see Materials and Methods). This analysis underlined that glycolysis is specifically sensitive mainly to GLUT, HK, and PGI (Figure 6).

Importantly, the structure of the metabolic network emerges from the clustering of the quantities illustrated in Figures 4 and 6: SA indexes referring to the variation of steady state fluxes of neighboring reactions are similar. This expected result is the consequence of having performed the SA at steady state, where mass conservation constrains the steady state fluxes of neighboring reactions according to their stoichiometry.

## Discussion

Several computational models have been proposed to study the control of the glycolytic flux. In Table 2 we summarized the main results obtained by those works that we based the definition of our model on. Marín-Hernández et al. (2006) performed an elasticity-based control analysis of the glycolytic flux in rat hepatoma cells (AS-30D); the authors observed that the major control of the glucose metabolism is due to the upstream part of glycolysis (GLUT, HK, GP, PGI, PFK). In a subsequent analysis based on a kinetic model, the same group calculated that glycolysis is mainly controlled by HK, HPI, and GLUT in AS-30D cells while glycogen degradation, GLUT and HK are key steps for HeLa cells. Using MCA, Schuster and Holzhutter (1995) calculated that the glycolytic flux in erythrocytes is mainly controlled by ATP utilization. Several computational studies have been done on the glycolysis of skeletal muscle cells. For example, Lambeth and Kushmerick (2002) identified in GP and PK the main steps of glycolytic control at resting flux; PFK and PGLM become important when the ATP utilization increases while GP activity gains importance in the network as the glycolytic flux increases.

**Table 2:** **Major controller of glycolysis reported in other computational and experimental works**.

Major controller of glucose flux	Experimental conditions	Reference
ATP utilization	Erythrocytes	Schuster and Holzhutter (1995)
ATPase, GP, PK, PFK, and PGLM	Skeletal muscle	Lambeth and Kushmerick (2002)
GLUT, HK, PGI, PFK, G6P branches	AS-30D	Marín-Hernández et al. (2006)
HK, HPI, GLUT	AS-30D	Marín-Hernández et al. (2011)
Glycogen degradation, GLUT, HK	HeLa	Marín-Hernández et al. (2011)

Comparison of the results obtained by different works should be done taking into account the structure of the metabolic network, the rate equations and the model configuration used. For example, a relevant control of the glycogen degradation step over the glycolytic flux was reported when glycogen was one of the sources of glucose for the system, as in Marín-Hernández et al. (2006) and Lambeth and Kushmerick, 2002 (Table 2). This was not the scenario of condition H, but, despite the differences in the metabolic network and metabolic state that we considered in comparison to Marín-Hernández et al. (2011), we also found that GLUT and HK exert a relevant control for glycolysis (Figure 6).

We compared two metabolic states generated by the specific variation of the fluxes regulated by the activity of the PI3K/Akt/mTOR pathway. One state represented the metabolism of a growing cancer cell characterized by aerobic glycolysis and cellular biosynthesis (condition H), while the other (condition L) represented the same metabolic network with a reduced glycolytic rate, a reduced lactic acid production, but a higher MPM, as reported in literature in relation to a lower activity of PI3K/Akt/mTOR (DeBerardinis et al., 2008). Some steps of the metabolic network that link glycolysis and PPP, namely those catalyzed by the G6PDH and TKL enzymes, revealed their importance for the cancer metabolic state. Results from our model may provide insight and assist in the selection of drug targets in anticancer treatments.

Results gained with current models in biology are still restricted by the assumptions made in relation to the limits of the current technologies, the lack of detailed information collected in the same experimental conditions and the need to simplify biological complexity (aspects as enzymes isoforms, molecular crowding and the temporal variation of enzyme concentration are usually not accounted for). Nevertheless, considering that model definition is related to the questions the modeler wants to answer, current models can be a useful tool for directing experimental efforts on a subset of all possible hypotheses. In relation to the crucial steps that emerged in this work for the dynamics of the metabolic network, it is worth to mention that, recently, several authors have recognized G6PDH and TKL as potential anticancer targets (e.g., Tennant et al., 2010; Vander Heiden, 2011). In general, there are several leading therapeutic compounds targeting glucose metabolism in preclinical and clinical phases for many tumor types, such as solid tumors (lung, breast, prostate, gastric), metastatic melanoma and renal cell carcinoma (Tennant et al., 2010; Porporato et al., 2011; Vander Heiden, 2011). Other principal targets that are currently enrolled in clinical and/or preclinical studies are: GLUT, HK, PK, LDH (preclinical and clinical; Mohanti et al., 1996; Singh et al., 2005; Christofk et al., 2008; Mathupala et al., 2009; Le et al., 2010; Wolf et al., 2011); PFK, PGYM, TKL, G6PDH (preclinical only; Kuo et al., 2000; Evans et al., 2005; Clem et al., 2008; Furuta et al., 2010).

It would be interesting to extend our analysis in order to compare other representative steady states, such as, for example, those considered by Sengupta et al. (2011), who evaluated different glucose metabolization patterns between glycolysis and PPP in growing and non-growing conditions. Moreover, our current model could be linked to a model of mitochondrion (e.g., Bazil et al., 2010) in order to include detailed representations of the tricarboxylic acid cycle and other relevant metabolic reactions that take place in the mitochondrion.

## Materials and Methods

### Numerical solutions

The DAE system representing the metabolic network was numerically integrated using MATLAB (2008b) and the stiff ode solver *ode15s* with absolute and relative tolerances of 10^−9^ and 10^−6^ respectively. Steady states were identified using the MATLAB function *fsolve* with default options. Model optimization and sensitivity analyses were done on HP(R) workstations equipped with two 2.50 GHz INTEL(R) Quad-core Xeon(R) E5420 processors and 10 GB RAM. The results obtained were displayed using MATLAB.

### Model optimization

Recently, it has been observed that multi-objective optimization have significant benefits compared to single objective approaches (Handl et al., 2007). Model fitting was formulated as a multi-objective optimization problem aiming at the simultaneous minimization of the difference between model predictions and experimentally determined concentrations, enzyme activities, and steady state fluxes. In detail, two objectives [*f*_1_(**x**), *f*_2_(**x**)] were defined as

$$f_{1,2}\left(\text{x}\right)=1∕N\Sigma_{i=1,\ldots ,N}\log_{10}\left(x_{i}∕x_{i}^{*}\right)$$

subject to

$$\begin{array}{llll} & {\text{J}}^{*}=\text{J}\left(\text{x}\right) & & \\ & \text{x}>0 & & \end{array}$$

where $x_{i}^{*}$ is the experimental value for the concentration of a metabolite (in the case of *f*_1_) or enzyme *V*_mf_ (for *f*_2_), *x*_i_ is the corresponding value used in the model, *N* is the number of elements (metabolites or enzymes), J* is the vector of experimental values of enzyme fluxes and **J**(**x**) are the respective model predictions obtained using **x**. The multi-objective optimization problem was solved using the Non-Dominated Sorting Genetic Algorithm II (Deb et al., 2002), which is one of the most popular methods in the field of multi-objective optimization. The NSGA-II algorithm was run in parallel using several populations of 1000 individuals for 100 generations. Every five generations, the worst five individuals of a population were replaced by a random selection of the best five individuals from the other populations.

### Modeling the metabolic effects of PI3K/Akt/mTOR

The metabolic effects of PI3K/Akt/mTOR were modeled according to the mechanism of interaction with its targets. Parameter values used to create condition L were obtained from condition H, multiplying a specific quantity appearing in the rate equation for the biochemical process regulated by a target of PI3K/Akt/mTOR by quantity α in order to reduce or increase the target activity. In detail, for GLUT, HK, PGI, GS, G6PDH, PGDH, TAL, TKL, TKL2, FBA, TPI, GAPDH, PGK, ENO, PK, LDH, and DPHase, we multiplied the respective *V*_f_ by α = 0.56, while for MPM we multiplied the respective *V*_f_ by α = 1.16; for PFK, we also multiplied the concentration of its allosteric activator F26P by α = 0.56. The *V*_f_ values used to obtain steady states H and L are listed in Table 1. Rate equations are listed in Appendix.

### Sensitivity analysis

Sensitivity analysis can be defined as the study of how uncertainty in the output of a model can be apportioned to different sources of uncertainty in the model input (Saltelli et al., 2000). In most of the current systems biology literature, sensitivities indexes are estimated calculating derivatives of a model output in a specific state of the system (local approach) corresponding to a particular model parameterization; moreover, only the variation of one parameter at a time is considered. For example, control coefficients estimated in the context of MCA are scaled partial derivatives calculated on the model linearized around a steady state; thus, MCA quantifies how a model output is influenced by infinitesimal changes in a parameter. As a consequence, results of MCA are restricted to infinitesimal parameter changes and do not account for interactions between parameters.

To overcome this issue, especially considering that the biological quantities represented with parameters (e.g., the enzyme concentration) are subject to significant variations, in our study we carried out the SA calculating the DGSMs (Kucherenko et al., 2009). The method is based on averaging local derivatives using (*quasi*) Monte Carlo (MC) sampling.

To compute the DGSMs we generated, for each of the conditions H and L, a set of parameterizations **p***_k_* = (*p*_*k*1_, *p*_*k*2_, … , *p_kn_*), *n* = 29, *k* = (1, 2, … , 500), **p***_k_* ∈ [**p** − ε**p**, **p** + ε**p**], where each **p***_k_* represents a possible perturbations of all the 29 *V*_f_ reference values (**p**) reported in Table 1. This sampling was carried out using the Sobol Sequences, a class of *quasi*-random low discrepancy sequences (Sobol, 1967). The parameterizations **p***_k_* were used to calculate the elementary effects

$$\begin{array}{llll} e^{ijk} & =\;\left|\right.\left[J_{i}\left(p_{k1},\ldots ,p_{kj}+\delta ,\ldots ,p_{kn}\right)-J_{i}\left(p_{k1},\ldots ,p_{kj},\ldots ,p_{kn}\right)\right]\left|\right./ & & \\ & \delta \cdot \left(p_{kj}∕\left|\right.J_{i}\left(p_{k1},\ldots ,p_{kj},\ldots ,p_{kn}\right)\left|\right.\right) & & \end{array}$$

a measure of the impact of having introduced the perturbation δ in the *j*-th element of **p***_k_* on the steady state flux *J_i_*, *i* = (1, 2, … , 29). Hence, for each of the two conditions H and L, we obtained a total of 500 29-by-29 matrices of elementary effects that were used to calculate the 29-by-29 matrices of scaled sensitivity indexes

$$g_{ij}=\left(m_{ij}^{2}+s_{ij}^{2}\right)/\sum_{j=1,\ldots ,29}\left(m_{ij}^{2}+s_{ij}^{2}\right)$$

where m*_ij_* and s*_ij_* are, respectively, the mean and sample standard deviation of the elementary effects *e_ijk_*.

The normalized differential ranking of the reactions was calculated as

$$R_{ij}=\left(r_{ij}^{L}-r_{ij}^{H}\right)/\left(r_{ij}^{L}+r_{ij}^{H}\right)$$

where *r_ij_* = (1, 2, … , 29) is the rank of the scaled sensitivity index *g_ij_* when ordering the elements (*g*_*i*1_, *g*_*i*2_, … , *g*_*i*29_) from the highest (strong influence of parameter *j* on flux *i*) to the lowest, and the superscript L or H indicates the condition. Hence, *R_ij_* will be positive (negative) for *V*_f_ parameters (and the corresponding reaction) exerting a higher (lower) control in condition H in relation to condition L; *R_ij_* will be higher for reactions having a higher control in both conditions.

The relative log sensitivity was calculated as

$$RLS_{ij}=log_{10}\left(g_{ij}/m_{i}^{\prime }\right)$$

and indicates the degree of variation of a scaled sensitivity index *g_ij_* in relation to the median *m_i_’* of all the scaled sensitivity indexes related to the same *V*_f_ parameter (*g*_1*j*_, *g*_2*j*_, … , *g*_29*j*_).

For each of the two conditions H and L, the 29-by-29 matrix of the first-order local sensitivity indexes *s_ij_* for steady state flux *i* to perturbation of *V*_f_ of reaction *j* was obtained by calculating the elementary effects *e_ijk_* using the respective values *V*_f_ listed in Table 1, that is **p***_k_* = **p**.

We used ε = 5 · 10^−6^ and δ = 10^−6^; steady state fluxes *J_i_* were obtained by numerical integration of the DAE system within the interval [0, 10^4^], followed by the identification of the steady state (see above the numerical solutions paragraph). For the calculation of the DGSMs, 2*N*(*n* + 1) numerical solutions of the model were required, where *N* = 500 represents the number of parameterizations, *n* = 29 is the number of the parameters, and the factor 2 is due to the two conditions H and L.

## Conflict of Interest Statement

The authors declare that the research was conducted in the absence of any commercial or financial relationships that could be construed as a potential conflict of interest.

## Appendix

### Differential algebraic equations

Here we report the list of equations that define the DAE system. *J_x_* denotes the flux of reaction or, more generally, biochemical process *x*; *V* is the volume of the homogeneous compartment in which all biochemical processes take place.

$$\begin{array}{llll} & \text{d AMP}/\text{dt}=0 & & \\ & \text{d ATP}/\text{dt}=\left(-J_{\text{HK}}+J_{\text{PK}}-J_{\text{ACL}}+J_{\text{cAK}}-J_{\text{ATPase}}-J_{\text{PFK}}\right.\left.+J_{\text{PGK}}-J_{\text{PRPPS}}+J_{\text{MPM}}-J_{\text{GS}}\right)∕V & & \\ & \text{d BPG}/\text{dt}=\left(J_{\text{GAPDH}}-J_{\text{PGK}}\right)/V & & \\ & \text{d CIT}/\text{dt}=0 & & \\ & \text{d DHAP}/\text{dt}=\left(J_{\text{FBA}}+J_{\text{TPI}}\right)/V & & \\ & \text{d E4P}/\text{dt}=\left(J_{\text{TAL}}-J_{\text{TKL2}}\right)/V & & \\ & \text{d F6P}/\text{dt}=\left(J_{\text{PGI}}-J_{\text{PFK}}+J_{\text{TKL2}}+J_{\text{TAL}}\right)/V & & \\ & \text{d F6P}/\text{dt}=\left(J_{\text{PGI}}-J_{\text{PFK}}+J_{\text{TKL2}}+J_{\text{TAL}}\right)/V & & \\ & \text{d F16P}/\text{dt}=\left(J_{\text{PFK}}-J_{\text{FBA}}\right)/V & & \\ & \text{d G1P}/\text{dt}=\left(J_{\text{GP}}-J_{\text{PGLM}}-J_{\text{GS}}\right)/V & & \\ & \text{d G6P}/\text{dt}=\left(J_{\text{HK}}-J_{\text{PGI}}-J_{\text{G6PDH}}+J_{\text{PGLM}}\right)/V & & \\ & \text{d GAP}/\text{dt}=\left(J_{\text{FBA}}-J_{\text{TPI}}-J_{\text{GAPDH}}+J_{\text{TKL}}+J_{\text{TKL2}}-J_{\text{TAL}}\right)/V & & \\ & \text{d GLC}/\text{dt}=\left(J_{\text{GLUT}}-J_{\text{HK}}\right)/V & & \\ & \text{d NAD}/\text{dt}=\left(J_{\text{LDH}}-J_{\text{GAPDH}}+J_{\text{DHase}}\right)/V & & \\ & \text{d NADP}/\text{dt}=\left(-J_{\text{G6PDH}}-J_{\text{PGDH}}+J_{\text{DPHase}}\right)/V & & \\ & \text{d Pi}/\text{dt}=0 & & \\ & \text{d PEP}/\text{dt}=\left(J_{\text{ENO}}-J_{\text{PK}}\right)/V & & \\ & \text{d PG2}/\text{dt}=\left(J_{\text{PGYM}}-J_{\text{ENO}}\right)/V & & \\ & \text{d PG3}/\text{dt}=\left(J_{\text{PGK}}-J_{\text{PGYM}}\right)/V & & \\ & \text{d PGN}/\text{dt}=\left(J_{\text{G6PDH}}-J_{\text{PGDH}}\right)/V & & \\ & \text{d PYR}/\text{dt}=\left(J_{\text{PK}}-J_{\text{LDH}}-\text{1}∕y\;J_{\text{MPM}}\right)/V & & \\ & \text{d LAC}/\text{dt}=0 & & \\ & \text{d R5P}/\text{dt}=\left(J_{\text{R5PI}}-J_{\text{TKL}}-J_{\text{PRPPS}}\right)/V & & \\ & \text{d RU5P}/\text{dt}=\left(J_{\text{PGDH}}-J_{\text{R5PI}}-J_{\text{RUPE}}\right)/V & & \\ & \text{d X5P}/\text{dt}=\left(J_{\text{RUPE}}-J_{\text{TKL}}-J_{\text{TKL2}}\right)/V & & \\ & \text{d S7P}/\text{dt}=\left(J_{\text{TKL}}-J_{\text{TAL}}\right)/V & & \end{array}$$

### Flux expressions

For each reaction, the corresponding flux expression and the parameter values are listed below with the references to the works they were taken from. When both the maximum rates of the forward (*V*_f_) and reverse (*V*_b_) reactions appear explicitly in the flux expression (e.g., see PGI), *V*_b_ was adjusted in agreement with the constraint imposed by the Haldane relationship (Sauro, 2011)

$$V_{\text{b}}=V_{\text{f}}\cdot K_{\text{mb}}/\left(K_{\text{eq}}\cdot K_{\text{mf}}\right)$$

on the basis of the optimized value of *V*_f_ (Table 1), the reactant constants for the forward (*K*_mf_) and backward (*K*_mb_) reaction, and the equilibrium constant (*K*_eq_).

#### Adenylate kinase

2ADP=ATP+AMP

Common modular rate law (Liebermeister et al., 2010).

$$\begin{array}{llll} \text{J}\text{\_}\text{AK} & =V_{\text{f}}\cdot \text{AD}{\text{P}}^{\text{2}}\cdot \left(\text{1}-\text{ATP}\cdot \text{AMP}/K_{\text{eq}}\right)/\left[{\left(\text{1}+\text{ADP}\right)}^{\text{2}}+\left(\text{1}+\text{ATP}\right)\cdot \left(\text{1}+\text{AMP}\right)-\text{1}\right] & & \end{array}$$

**Table:**

Parameter	Value	Unit	Reference
*K*_eq_	2.26	–	Marín-Hernández et al. (2011)

#### Consumption of ATP (ATPase)

ATP→ADP+Pi

Common modular rate law (Liebermeister et al., 2010).

J_ATPase=Vf⋅ATP

#### Oxidation of NADH (DHase)

NADH=NAD+H

Common modular rate law (Liebermeister et al., 2010).

$$\begin{array}{llll} \text{J}\text{\_}\text{DHase} & =V_{\text{f}}\cdot \text{NADH}\cdot \left[\text{1}-\text{NAD}/\left(\text{NADH}\cdot K_{\text{eq}}\right)\right]∕\left[\left(\text{1}+\text{NADH}\right)+\left(\text{1}+\text{NAD}\right)-\text{1}\right] & & \end{array}$$

**Table:**

Parameter	Value	Unit	Reference
*K*_eq_	300	–	Marín-Hernández et al. (2011) used 250

#### Oxidation of NADPH (DPHase)

NADPH=NADP+H

Common modular rate law (Liebermeister et al., 2010).

$$\begin{array}{llll} \text{J}\text{\_}\text{DPHase} & =V_{\text{f}}\cdot \text{NADPH}\cdot \left[\text{1}-\text{NADP}/\left(\text{NADPH}\cdot K_{\text{eq}}\right)\right]/\left[\left(\text{1}+\text{NADPH}\right)+\left(\text{1}+\text{NADP}\right)-\text{1}\right] & & \end{array}$$

**Table:**

Parameter	Value	Unit	Reference
*K*_eq_	0.2	–	Adjusted

#### Enolase (ENO)

PG2=PEP

The rate equation is from Marín-Hernández et al. (2011).

$$V_{\text{mr}}=V_{\text{mf}}/K_{\text{eq}}\cdot K_{\text{mp}}/K_{\text{ms}}$$

**Table:**

Parameter	Value	Unit	Reference
*K*_ms_	0.038e-3	M	Marín-Hernández et al. (2011)
*K*_mp_	0.06e-3	M	Marín-Hernández et al. (2011)
*K*_eq_	1.4127	–	Calculated from Marín-Hernández et al. (2011)

#### Fructose bisphosphate aldolase

F16P=GAP+DHAP

Flux expression from Marín-Hernández et al. (2011).

$$\begin{array}{l} V_{\text{mr}}=V_{\text{mf}}/K_{\text{eq}}\cdot (K_{\text{dhap}}\cdot K_{\text{g}3\text{p}}/K_{\text{fbp}})\\ A=\text{F}16\text{P};\;P=\text{DHAP};Q=\text{GAP};\\ {\text{J}}_{\text{FBA}}=(V_{\text{mf}}\cdot A/K_{\text{fbp}}-V_{\text{mr}}\cdot P\cdot Q/(K_{\text{dhap}}\cdot K_{\text{g}3\text{p}}))/\left(1+A/K_{\text{fbp}}+P/K_{\text{dhap}}+Q/K_{g3p}+P\cdot Q/(K_{\text{dhap}}\cdot K_{g3p})\right) \end{array}$$

**Table:**

Parameter	Value	Unit	Reference
*K*_fbp_	0.009e-3	M	Marín-Hernández et al. (2011)
*K*_dhap_	0.08e-3	M	Marín-Hernández et al. (2011)
*K*_g3p_	0.16e-3	M	Marín-Hernández et al. (2011)
*K*_eq_	0.0018	–	Calculated from Marín-Hernández et al. (2011)

#### Glyceraldehyde-3-phosphate dehydrogenase

GAP+NAD+Pi=BPG+NADH+H

Flux expression from Marín-Hernández et al. (2011).

$$\begin{array}{ll} A & =\text{NAD};\;B=\text{GAP};\;C=\text{Pi};\;P=\text{BPG};\;Q=\text{NADH};\\ V_{\text{mr}} & =V_{\text{mf}}/K_{\text{eq}}\cdot K_{\text{dpg}}\cdot K_{\text{nadh}}/\left(K_{\text{g3p}}\cdot K_{\text{nad}}\cdot K_{\text{p}}\right);\\ \text{J}\text{\_}\text{GAPDH} & =\left(V_{\text{mf}}\cdot A\cdot B\cdot C/\left(K_{\text{nad}}\cdot K_{\text{g3p}}\cdot K_{\text{p}}\right)\right.\left.-V_{\text{mr}}\cdot P\cdot Q/\left(K_{\text{dpg}}\cdot K_{\text{nadh}}\right)\right)/\left(\text{1}+A/K_{\text{nad}}+A\cdot B/\right.\left(K_{\text{nad}}\cdot K_{\text{g3p}}\right)\\ & \;+A\cdot B\cdot C/\left(K_{\text{nad}}\cdot K_{\text{g3p}}\cdot K_{\text{p}}\right)+P\cdot Q/\left.\left(K_{\text{dpg}}\cdot K_{\text{nadh}}\right)+Q/K_{\text{nadh}}\right) \end{array}$$

**Table:**

Parameter	Value	Unit	Reference
*K*_nad_	0.09e-3	M	Marín-Hernández et al. (2011)
*K*_g3p_	0.19e-3	M	Marín-Hernández et al. (2011)
*K*_p_	29e-3	M	Marín-Hernández et al. (2011)
*K*_dpg_	0.022e-3	M	Marín-Hernández et al. (2011)
*K*_nadh_	0.01e-3	M	Marín-Hernández et al. (2011)
*K*_eq_	0.3574	–	Calculated from Marín-Hernández et al. (2011)

#### Glucose-6-phosphate dehydrogenase

As in Holzhütter (2004) we used the sum of the consecutive reactions G6P + NADP + PGLT + NADPH + H and PGLT + PGN

G6P+NADP=PGN+NADPH+H

Flux expression from Holzhütter (2004).

$$\begin{array}{l} \text{J\_G6PDH}=V_{\max}/K_{\text{G6P}}/K_{\text{NADP}}\cdot \left(\text{G6P}\cdot \text{NADP}-\text{PGN}\cdot \text{NADPH}/K_{\text{app}}\right)/(1+\text{NADP}\cdot \left(1+\text{G6P}/K_{\text{G6P}}\right)\\ \;\;/K_{\text{NADP}}+\text{ATP}/K_{\text{ATP}}+\text{NADPH}/K_{\text{NADPH}}+\text{BPG}/K_{\text{PGA23}}) \end{array}$$

**Table:**

Parameter	Value	Unit	Reference
*K*_app_	2000	–	Holzhütter (2004)
*K*_G6P_	0.0667e-3	mM	Holzhütter (2004)
*K*_NADP_	0.00367e-3	mM	Holzhütter (2004)
*K*_ATP_	0.749e-3	mM	Holzhütter (2004)
*K*_NADPH_	0.00312e-3	mM	Holzhütter (2004)
*K*_PGA23_	2.289e-3	mM	Holzhütter (2004)

#### Glucose transport

GLCe=GLC

Monosubstrate reversible Michaelis–Menten equation (Marín-Hernández et al., 2011).

$$\begin{array}{llll} & J\text{\_}\text{glut}=V_{\max f}\cdot \left(\text{GLC}\text{\_}\text{e}-\text{GLC}∕k_{\text{eq}}\right)∕\left(K\text{Glc}\text{\_}\text{e}\cdot \left(\text{1}+\text{GLC}∕K_{\text{Glc}}\right)+\text{GLC}\text{\_}\text{e}\right) & & \end{array}$$

**Table:**

Parameter	Value	Unit	Reference
*k*_eq_	1	–	Marín-Hernández et al. (2011)
*K*_Glc_	9.3e-3	M	Marín-Hernández et al. (2011)
*K*_Glc_e_	10e-3	M	Marín-Hernández et al. (2011)

#### Glycogen Phosphorylase A

GLYn+Pi=GLYm+G1P

Flux expression from Lambeth and Kushmerick (2002), where GLYn = GLYm = GLY.

$$\begin{array}{ll} V_{\max r} & =V_{\max f}\cdot K_{\text{iGLYb}}\cdot K_{\text{iG1P}}∕\left(K_{\text{iGLY}f}\cdot K_{\text{Pi}}\cdot K_{\text{eq}}\right)\\ J\text{\_}\text{GPa} & =\left(V_{\text{max }f}\cdot \left(\text{GLY}\cdot \text{Pi}∕\left(K{\text{i}}_{\text{GLYf}}\cdot K\text{Pi}\right)\right)\right.\left.-V_{\text{max}r}\cdot \left(\text{GLY}\cdot \text{G1P}∕\left(K_{\text{GLYb}}\cdot K{\text{i}}_{\text{G1P}}\right)\right)\right)\\ & \;∕\left(\text{1}+\text{GLY}∕K{\text{i}}_{\text{GLY}f}+\text{Pi}∕K\text{iPi}+\text{GLY}∕K{\text{i}}_{\text{GLYb}}+\text{G1P}∕K{\text{i}}_{\text{G1P}}\right)\left.+\text{GLY}\cdot \text{Pi}∕\left(K{\text{i}}_{\text{GLYf}}\cdot K\text{iPi}\right)+\left(\text{GLY}\cdot \text{G1P}\right)∕\left(K{\text{i}}_{\text{GLYb}}\cdot K{\text{i}}_{\text{G1P}}\right)\right) \end{array}$$

**Table:**

Parameter	Value	Unit	Reference
K_GLYf_	1.7e-3	M	Lambeth and Kushmerick (2002)
K_Pi_	4e-3	M	Lambeth and Kushmerick (2002)
K_iGLY_	0.2e-3	M	Lambeth and Kushmerick (2002)
K_iPi_	4.7e-3	M	Lambeth and Kushmerick (2002)
*K*_GLYb_	0.15e-3	M	Lambeth and Kushmerick (2002)
*K*_G1P_	2.7e-3	M	Lambeth and Kushmerick (2002)
K_iG1P_	10.1e-3	M	Lambeth and Kushmerick (2002)
*K*_eq_	0.42	–	Lambeth and Kushmerick (2002)

#### Glycogen Phosphorylase B

GLYn+Pi=GLYm+G1P

Flux expression from Lambeth and Kushmerick (2002), where GLYn=GLYm=GLY.

$$\begin{array}{l} J\_\text{GPb}=\left(V_{\max f}\cdot \left(\text{GLY}\cdot \text{Pi}/K{\text{i}}_{\text{GLYf}}\cdot K\text{Pi}\right)\right)-V_{\max r}\left(\text{GLY}\cdot \text{G1P}/\left(K{\text{i}}_{\text{GLYb}}\cdot K_{\text{G1P}}\right)\right))\\ \;/\left(1+\text{GLY}/K{\text{i}}_{\text{GLYf}}+\text{Pi}/K\text{iPi}+\text{GLY}/K{\text{i}}_{\text{GLYb}}+\text{G1P}/K{\text{i}}_{\text{G1P}}+\left(\text{GLY}\cdot \text{Pi}\right)/\left(K{\text{i}}_{\text{GLYf}}\cdot K\text{Pi}\right)+\left(\text{GLY}\cdot \text{G1P}\right)/\left(K{\text{i}}_{\text{GLYb}}\cdot K_{\text{G1P}}\right)\right)\\ \;\cdot \left({\text{AMP}}^{\text{nH}}/K_{\text{amp}}\right)/\left({\text{1+AMP}}^{\text{nH}}/K_{\text{amp}}\right); \end{array}$$

**Table:**

Parameter	Value	Unit	Reference
*K*_Pi_	0.2e-3	M	Lambeth and Kushmerick (2002)
*K*_iPi_	4.6e-3	M	Lambeth and Kushmerick (2002)
*K*_iGLYf_	15e-3	M	Lambeth and Kushmerick (2002)
*K*_G1P_	1.5e-3	M	Lambeth and Kushmerick (2002)
*K*_iG1P_	7.4e-3	M	Lambeth and Kushmerick (2002)
*K*_iGLYb_	4.4e-3	M	Lambeth and Kushmerick (2002)
nH	1.75	–	Lambeth and Kushmerick (2002)
*K*_amp_	1.9e-12	M	Lambeth and Kushmerick (2002)
*K*_eq_	16.62	–	Lambeth and Kushmerick (2002)

#### Glycogen synthase

We used the sum of three reactions G1P+UTP=UDP-Glc+2Pi,UDP-Glc+GLYn=UDP+GLYm and UDP+ATP=UTP+ ADP

GLY+G1P+ATP=GLY+ADP+2Pi

Flux expression from Li et al. (2010b).

$$\begin{array}{ll} \text{GLYn} & =\text{GLYm}=\text{GLY}\\ J\text{\_}\text{GS} & =V_{\text{maxf}}∕K_{\text{f}}\cdot \text{G1P}\cdot \text{ATP}\cdot \text{GLY}\cdot \left(\text{1}-\text{P}{\text{i}}^{\text{2}}\cdot \text{ADP/(G1P}\cdot \text{ATP}\cdot K_{\text{eq}}\text{)}\right)∕\text{(1 + G1P}\cdot \text{ATP}\cdot \text{GLY/}K_{\text{f}}\text{ + GLY}\cdot \text{P}{\text{i}}^{\text{2}}\cdot \text{ADP/}K_{\text{r}}\text{)} \end{array}$$

**Table:**

Parameter	Value	Unit	Reference
*K*_f_	1.740e-04	–	Li et al. (2010b)
*K*_r_	1.580e-02	–	Li et al. (2010b)
*K*_eq_	2.671e-05	–	Li et al. (2010b)

#### Hexokinase

GLC+ATP=ADP+G6P

Random bi-substrate Michaelis–Menten (Marín-Hernández et al., 2011).

$$\begin{array}{ll} A & =\text{GLC};\;B=\text{ATP};\;P=\text{G6P};\;Q=\text{ADP}\\ J\text{\_}\text{HK} & =\left(V_{\text{mf}}∕\left(K_{\text{a}}\cdot K_{\text{b}}\right)\cdot \left(A\cdot B-P\cdot Q∕K_{\text{app}}\right)\right)∕\left(\text{1}+A∕K_{\text{a}}+B∕K_{\text{b}}+A\cdot B∕\left(K_{\text{a}}\cdot K_{\text{b}}\right)+P∕K_{\text{p}}+Q∕K_{\text{q}}+P\cdot Q\right.\\ & \left.\;∕\left(K_{\text{p}}\cdot K_{\text{q}}\right)+A\cdot Q∕\left(K_{\text{a}}\cdot K_{\text{q}}\right)+P\cdot B∕\left(K_{\text{p}}\cdot K_{\text{b}}\right)\right) \end{array}$$

**Table:**

Parameter	Value	Unit	Reference
*K*_a_	0.1	mM	Marín-Hernández et al. (2011)
*K*_b_	1.1	mM	Marín-Hernández et al. (2011)
*K*_p_	0.02	mM	Marín-Hernández et al. (2011)
*K*_q_	3.5	mM	Marín-Hernández et al. (2011)
*K*_app_	651	–	Marín-Hernández et al. (2011)

#### Lactate dehydrogenase

PYR+NADH=LAC+NAD

The rate equation is from Marín-Hernández et al. (2011).

$$\begin{array}{ll} A & =\text{NADH},\;B=\text{PYR},\;P=\text{LAC},\;Q=\text{NAD}\\ V_{\text{mr}} & =V_{\text{mf}}∕K_{\text{eq}}\cdot K_{\text{p}}\cdot {\text{K}}_{\text{q}}∕\left(K_{\text{a}}\cdot K_{\text{b}}\right)\\ J_{\text{LDH}} & =\left(V_{\text{mf}}\cdot A\cdot B∕\left(\text{alfa}\cdot K_{\text{a}}\cdot K_{\text{b}}\right)-V_{\text{mr}}\cdot P\cdot Q∕\left(\text{beta}\cdot K_{\text{p}}\cdot K_{\text{q}}\right)\right)\\ & \;∕\left(\text{1}+A∕K_{\text{a}}+B∕K_{\text{b}}+A\cdot B∕\left(\text{alfa}\cdot K_{\text{a}}\cdot K_{\text{b}}\right)+P\cdot Q∕\left(\text{beta}\cdot K_{\text{p}}\cdot K_{\text{q}}\right)+P∕K_{\text{p}}+Q∕K_{\text{q}}\;\right); \end{array}$$

**Table:**

Parameter	Value	Unit	Reference
alfa	1	–	Marín-Hernández et al. (2011)
*K*_a_	0.002e-3	M	Marín-Hernández et al. (2011)
*K*_b_	0.3e-3	M	Marín-Hernández et al. (2011)
beta	1	-	Marín-Hernández et al. (2011)
*K*_p_	4.7e-3	M	Marín-Hernández et al. (2011)
*K*_q_	0.07e-3	M	Marín-Hernández et al. (2011)
*K*_eq_	3.4525e+03	–	Calculated from Marín-Hernández et al. (2011)

#### Mitochondrial pyruvate metabolism

Common modular rate law (Liebermeister et al., 2010).

$$\text{1}∕y\text{ PYR}+\text{5}∕\left(\text{2}y\right)\text{O}2+\text{Pi}+\text{ADP}\to \text{3}∕y\text{ CO2}+\text{5}∕y\;{\text{H}}_{\text{2}}\text{O}+\text{ATP}$$

$$\begin{array}{l} J_{\text{M}}\text{PM}=V_{\text{f}}\cdot ({\text{PYR}}^{1/\text{y}}\cdot \text{Pi}\cdot \text{ADP}\cdot O_{2}^{5/\left(2\cdot y\right)}\cdot \left[1-\left(\text{ATP}\cdot {\text{CO}}_{2}^{\text{3}/y}\right)/\left({\text{PYR}}^{1/y}\cdot {\text{O}}_{2}^{5/\left(2\cdot y\right)}\cdot \text{Pi}\cdot \text{ADP}\cdot K\text{eq}\right)\right]\\ \;\;/\left[{\left(1+\text{PYR}\right)}^{1/y}\cdot {\left(1+{\text{O}}_{2}\right)}^{5/\left(2\cdot y\right)}\cdot \left(1+\text{Pi}\right)\cdot \left(1+\text{ADP}\right)+\left(1+\text{ADP}\right)\cdot {\left(1+{\text{CO}}_{\text{2}}\right)}^{3/y}-1\right] \end{array}$$

**Table:**

Parameter	Value	Unit	Reference
*K*_eq_	106	–	Adjusted
*y*	12.5	–	Commonly accepted average ATP yield

#### Phosphofructokinase

F6P+ATP=F16P+ADP

Flux expression from Marín-Hernández et al. (2011).

A=F6P; B=ATP; P=F16P; Q=ADP.

$${\text{J}}_{\text{PFK}}=\left(\begin{array}{c} V_{\text{m}}\cdot \left(B/K_{\text{atp}}\right)/\left(1+\left(B/K_{\text{atp}}\right)\right)\cdot \left(1+\left(\text{beta}\cdot \text{F26P}\right)/\left(\text{alfa}\cdot \text{Kf26bp}\right)\right)/\left(1+\left(\text{F26P}/\left(\text{alfa}\cdot \text{Kf26bp}\right)\right)\right)\cdot \\ \left(A\cdot \left(1+\left(\text{F26P}/\left(\text{alfa}\cdot \text{Kf26bp}\right)\right)\right)\right)/\left(\text{Kf6p}\cdot \left(\text{1+}\left(\text{F26P}/\text{Kf26bp}\right)\right)\right)\cdot \left(1+\left(A\cdot \left(1+\left(\text{F26P}/\left(\text{alfa}\cdot \text{Kf26bp}\right)\right)\right)\right)\right)/\;\\ {\left(\text{Kf6p}\cdot \left(1+\left(\text{F26P}/\text{Kf26bp}\right)\right)\right)}^{3}/\left(L\cdot {\left(1+\text{CIT}/K_{cit}\right)}^{4}\cdot {\left(1+B/K_{\text{iatp}}\right)}^{4}\right)/{\left(1+\left(\text{F26P}/\text{Kf26bp}\right)\right)}^{4}\\ +\left(1+\left(A\cdot \left(1+\left(\text{F26P}/\left(\text{alfa}\cdot \text{Kf26bp}\right)\right)\right)\right)\right)/{\left(\text{Kf6p}\cdot \left(1+\left(\text{F26P}/\text{Kf26bp}\right)\right)\right)}^{4}-(\left(Q\cdot P\right)/\left(K_{\text{adp}}\cdot K_{\text{fbp}}\cdot K_{\text{app}}\right)/\;\\ \left(\text{Q}/K_{\text{adp}}\right)+\left(P/K_{\text{fbp}}\right)+\left(\left(Q\cdot P\right)/\left(K_{\text{adp}}\cdot K_{\text{fbp}}\right)+1\right) \end{array}\right)$$

**Table:**

Parameter	Value	Unit	Reference
*K*_atp_	0.021	mM	Marín-Hernández et al. (2011)
beta	0.98	–	Marín-Hernández et al. (2011)
alfa	0.32	–	Marín-Hernández et al. (2011)
*K*_f26bp_	0.00084	mM	Marín-Hernández et al. (2011)
*K*_f6p_	1.0	mM	Marín-Hernández et al. (2011)
*L*	4.1	mM	Marín-Hernández et al. (2011)
*K*_cit_	6.8	mM	Marín-Hernández et al. (2011)
Ki_atp_	20	mM	Marín-Hernández et al. (2011)
*K*_adp_	5	mM	Marín-Hernández et al. (2011)
*K*_fbp_	5	mM	Marín-Hernández et al. (2011)
*K*_app_	247	–	Marín-Hernández et al. (2011)

#### Phosphogluconate dehydrogenase

PGN+NADP=RU5P+CO2+NADPH+2H

Flux expression from Holzhütter (2004).

$$\begin{array}{l} J_{\text{PGDH}}=V_{\max}/K_{\text{6PGI}}/K_{\text{NADP}}\cdot \left(\text{PGN}\cdot \text{NADP}-\text{RU5P}\cdot \text{NADPH}/K_{\text{app}}\right)/(\left(1+\text{NADP}/K_{\text{NADP}}\right)\\ \;\cdot \left(1+\text{PGN}/K_{\text{6PG1}}+\text{BPG}/K_{\text{PGA23}}\right)+\text{ATP}/K_{\text{ATP}}+\text{NADPH}\cdot \left(\text{1+}\text{PGN}/K_{\text{6PG2}}\right)/K_{\text{NADPH}}) \end{array}$$

**Table:**

Parameter	Value	Unit	Reference
*K*_app_	141.7	–	Holzhütter (2004)
*K*_6PG1_	0.01e-3	mM	Holzhütter (2004)
*K*_6PG2_	0.058e-3	mM	Holzhütter (2004)
*K*_PGA23_	0.12e-3	mM	Holzhütter (2004)
*K*_NADP_	0.018e-3	mM	Holzhütter (2004)
*K*_NADPH_	0.0045e-3	mM	Holzhütter (2004)
*K*_ATP_	0.154e-3	mM	Holzhütter (2004)

#### Phosphoglucoisomerase

Monoreactant reversible equation with competitive inhibition by E4P, 6PG, and FBP (Marín-Hernández et al., 2011).

$$\begin{array}{ll} \text{G6P} & =\text{F6P}\\ V_{\text{mr}} & =V_{\text{mf}}\cdot K_{\text{f6p}}∕\left(K_{\text{eq}}\cdot K_{\text{g6p}}\right)\\ J_{\text{PGI}} & =\left(V_{\text{mf}}\cdot \text{G6P}∕K_{\text{g6p}}-V_{\text{mr}}\cdot \text{F6P}∕K_{\text{f6p}}\right)∕\left(\text{1}+A∕K_{\text{g6p}}+P∕K_{\text{f6p}}+\text{E4P}∕K_{\text{ery4p}}+\text{F16P}∕K_{\text{fbp}}+\text{PGN}∕K_{\text{pg}}\right) \end{array}$$

**Table:**

Parameter	Value	Unit	Reference
*K*_g6p_	0.4e-3	M	Marín-Hernández et al. (2011)
*K*_f6p_	0.05e-3	M	Marín-Hernández et al. (2011)
*K*_ery4p_	0.001e-3	M	Marín-Hernández et al. (2011)
*K*_fbp_	0.06e-3	M	Marín-Hernández et al. (2011)
*K*_pg_	0.015e-3	M	Marín-Hernández et al. (2011)
*K*_eq_	5.56e-2	–	Calculated from Marín-Hernández et al. (2011)

#### Phosphoglucomutase

G1P=G6P

Flux expression from Lambeth and Kushmerick (2002).

$$\begin{array}{ll} V_{\text{maxr}} & =V_{\text{maxf}}\cdot K_{\text{G6P}}∕\left(K_{\text{G1P}}\cdot K_{\text{eq}}\right)\\ J_{\text{PGLM}} & =\left(V_{\text{maxf}}\cdot \text{G1P}∕K_{\text{G1P}}-V_{\text{maxr}}\cdot \text{G6P}∕K_{\text{G6P}}\right)\;∕\;\left(\text{ 1}+\text{G1P}∕K_{\text{G1P}}+\text{G6P}∕K_{\text{G6P}}\right) \end{array}$$

**Table:**

Parameter	Value	Unit	Reference
*K*_eq_	17.2	–	Gao and Leary (2004)
*K*_G1P_	0.063e-3	M	Lambeth and Kushmerick (2002)
*K*_G6P_	0.03e-3	M	Lambeth and Kushmerick (2002)

#### Phosphoglycerate kinase

BPG+ADP=PG3+ATP

Flux expression from Marín-Hernández et al. (2011).

$$\begin{array}{ll} A & =\text{BPG},\;B=\text{ADP},\;P=\text{PG3},\;Q=\text{ATP}\\ V_{\text{mr}} & =V_{\text{mf}}∕K_{\text{eq}}\cdot K_{\text{p}}\cdot K_{\text{q}}∕\left(K_{\text{a}}\cdot K_{\text{b}}\right)\\ J_{\text{PGK}} & =\left(V_{\text{mf}}\cdot A\cdot B∕\left(\text{alfa}\cdot K_{\text{a}}\cdot K_{\text{b}}\right)-V_{\text{mr}}\cdot P\cdot Q∕\left(\text{beta}\cdot K_{\text{p}}\cdot K_{\text{q}}\right)\right)\\ & \;∕\left(\text{1}+A∕K_{\text{a}}+B∕K_{\text{b}}+A\cdot B∕\left(\text{alfa}\cdot K_{\text{a}}\cdot K_{\text{b}}\right)+P\cdot Q∕\left(\text{beta}\cdot K_{\text{p}}\cdot K_{\text{q}}\right)+P∕K_{\text{p}}+Q∕K_{\text{q}}\;\right) \end{array}$$

**Table:**

Parameter	Value	Unit	Reference
Alfa	1	–	Marín-Hernández et al. (2011)
*K*_a_	0.079e-3	M	Marín-Hernández et al. (2011)
*K*_b_	0.04e-3	M	Marín-Hernández et al. (2011)
Beta	1	–	Marín-Hernández et al. (2011)
*K*_p_	0.13e-3	M	Marín-Hernández et al. (2011)
*K*_q_	0.27e-3	M	Marín-Hernández et al. (2011)
*K*_eq_	11.369	–	Calculated from Marín-Hernández et al. (2011)

#### Pyruvate kinase

PEP+ADP=PYR+ATP

The rate equation is from Marín-Hernández et al. (2011).

$$\begin{array}{l} {\text{J}}_{\text{PK}}=V_{\text{m}}\cdot \left(B/K_{\text{adp}}\right)/\left(1+\left(B/K_{\text{adp}}\right)\right)\cdot \left(\left(A/K_{\text{pep}}\right)\cdot {\left(1+\left(A/K_{\text{pep}}\right)\right)}^{3}\right)/(\left(\left(L\cdot {\left(1+\left(Q/K_{\text{iatp}}\right)\right)}^{4}\right)/{\left(1+\left(\text{F16P}/K_{\text{fbp}}\right)\right)}^{4}\right)\\ \;+{\left(1+\left(A/K_{\text{pep}}\right)\right)}^{4})-\left(\left(Q\cdot P\right)/\left(K_{\text{atp}}\cdot K_{\text{pyr}}\cdot K_{\text{app}}\right)\right)/\left(\left(\left(Q/K_{\text{atp}}\right)+\left(P/K_{\text{pyr}}\right)\right)+\left(\left(Q\cdot P\right)/\left(K_{\text{atp}}\cdot K_{\text{pyr}}\right)\right)+1\right)); \end{array}$$

**Table:**

Parameter	Value	Unit	Reference
*K*_adp_	0.4	mM	Marín-Hernández et al. (2011)
*K*_pep_	0.014	mM	Marín-Hernández et al. (2011)
*L*	1	–	Marín-Hernández et al. (2011)
*K*_iatp_	2.5	mM	Marín-Hernández et al. (2011)
*K*_fbp_	0.0004	mM	Marín-Hernández et al. (2011)
*K*_atp_	0.86	mM	Marín-Hernández et al. (2011)
*K*_pyr_	10	mM	Marín-Hernández et al. (2011)
*K*_app_	195172	–	Marín-Hernández et al. (2011)

#### Phosphoribosylpyrophosphate synthetase

R5P+ATP=AMP+PRPP

Flux expression from Holzhütter (2004).

$$\begin{array}{c} J_{\text{PRPPS}}=V_{\text{max}}\cdot \left(\text{R5P}\cdot \text{ATP}-\text{PRPP}\cdot \text{AMP}∕K_{\text{app}}\right)∕\left(\left(K_{\text{ATP}}+\text{ATP}\right)\cdot \left(K_{\text{R5P}}+\text{R5P}\right)\right) \end{array}$$

**Table:**

Parameter	Value	Unit	Reference
*K*_app_	1e5	–	Holzhütter (2004)
*K*_ATP_	0.0300e-3	mM	Holzhütter (2004)
*K*_R5P_	0.57e-3	mM	Holzhütter (2004)

#### Phosphoglycerate mutase

PG3=PG2

The rate equation is from Marín-Hernández et al. (2011).

$$\begin{array}{ll} V_{\text{mr}} & =V_{\text{mf}}∕K_{\text{eq}}\cdot K_{\text{mp}}∕K_{\text{ms}}\\ J_{\text{PGYM}} & =\left(V_{\text{mf}}\cdot \text{PG3}∕K_{\text{ms}}-V_{\text{mr}}\cdot \text{PG2}∕K_{\text{mp}}\right)∕\left(\text{1}+\text{PG3}∕K_{\text{ms}}+\text{PG2}∕K_{\text{mp}}\right); \end{array}$$

**Table:**

Parameter	Value	Unit	Reference
*K*_ms_	0.19e-3	M	Marín-Hernández et al. (2011)
*K*_mp_	0.12e-3	M	Marín-Hernández et al. (2011)
*K*_eq_	1.6491	–	Calculated from Marín-Hernández et al. (2011)

#### Ribose-5-phosphate isomerase

RU5P=R5P

Flux expression from Schuster and Holzhutter (1995).

$$\begin{array}{c} J_{\text{R5PI}}=V_{\text{max}}\cdot \left(\text{RU5P}-\text{R5P}∕{K_{\text{eq}}}_{\text{R5PI}}\right)∕\left(\text{RU5P}+K_{\text{Ru5P}}\cdot \left(\text{1}+\text{R5P}∕K_{\text{R5P}}\right)\right) \end{array}$$

**Table:**

Parameter	Value	Unit	Reference
*K*_eq_R5PI_	3	–	Holzhütter (2004)
*K*_Ru5P_	0.78e-3	mM	Holzhütter (2004)
*K*_R5P_	2.2e-3	mM	Holzhütter (2004)

#### Ribulose-phosphate epimerase

RU5P=X5P

Flux expression from Holzhütter (2004).

$$\begin{array}{c} J_{\text{RUPE}}=V_{\text{max}}\cdot \left(\text{RU5P}-\text{X5P}∕{K_{\text{eq}}}_{\text{RUPE}}\right)∕\left(\text{RU5P}+K_{\text{Ru5P}}\cdot \left(\text{1}+\text{X5P}∕K_{\text{X5P}}\right)\right) \end{array}$$

**Table:**

Parameter	Value	Unit	Reference
*K*_eq_RUPE_	2.7	–	Holzhütter (2004)
*K*_Ru5P_	0.19e-3	mM	Holzhütter (2004)
*K*_X5P_	0.5e-3	mM	Holzhütter (2004)

#### Transaldolase

S7P+GAP=F6P+E4P

Flux expression from Schuster and Holzhutter (1995).

$$\begin{array}{l} J_{\text{TAL}}=V_{\max}\cdot \left(\text{S7P}\cdot \text{GAP}-\text{E4P}\cdot \text{F6P}/K_{\text{eqTAL}}\right)/(\left(K_{1}+\text{GAP}\right)\cdot \text{S7P}+\left(K_{2}+K_{6}\cdot \text{F6P}\right)\cdot \text{GAP}+\left(K_{3}+K_{5}\cdot \text{F6P}\right)\cdot \text{E4P}\\ \;+K_{4}\cdot \text{F6P}+K_{7}\cdot \text{S7P}\cdot \text{E4P}) \end{array}$$

**Table:**

Parameter	Value	Unit	Reference
*K*_1_	0.00823e-3	mM	Holzhütter (2004)
*K*_2_	0.04765e-3	mM	Holzhütter (2004)
*K*_3_	0.1733e-3	mM	Holzhütter (2004)
*K*_4_	0.006095e-3	mM	Holzhütter (2004)
*K*_5_	0.8683	–	Holzhütter (2004)
*K*_6_	0.4653	–	Holzhütter (2004)
*K*_7_	2.524	–	Holzhütter (2004)
*K*_eq_TKL2_	2.703	–	Casazza and Veech (1986)

#### Transketolase

R5P+X5P=GAP+S7P

Flux expression from Schuster and Holzhutter (1995).

$$\begin{array}{l} J_{\text{TKL}}=V_{\max}\cdot \left(\text{R5P}\cdot \text{X5P}-\text{GAP}\cdot \text{S7P}/K_{\text{eqTKL}}\right)/(\left(K_{1}+\text{R5P}\right)\cdot X5\text{P}+\left(K_{2}+K_{6}\cdot \text{S}7\text{P}\right)\cdot \text{R5P}+\left(K_{3}+K_{5}\cdot \text{S}7\text{P}\right)\cdot \text{GAP}\\ \;+K_{4}\cdot \text{S7P}+K_{7}\cdot \text{X5P}\cdot \text{GAP}) \end{array}$$

**Table:**

Parameter	Value	Unit	Reference
*K*_1_	0.4177e-3	mM	Holzhütter (2004)
*K*_2_	0.3055e-3	mM	Holzhütter (2004)
*K*_3_	12.432e-3	mM	Holzhütter (2004)
*K*_4_	0.00496e-3	mM	Holzhütter (2004)
*K*_5_	0.41139	–	Holzhütter (2004)
*K*_6_	0.00774	–	Holzhütter (2004)
*K*_7_	48.8	–	Holzhütter (2004)
*K*_eq_TKL_	2.08	–	Casazza and Veech (1986)

#### Transketolase 2

X5P+E4P=GAP+F6P

Flux expression from Schuster and Holzhutter (1995).

$$\begin{array}{l} J_{\text{TKL2}}=V_{\max}\cdot \left(\text{E4P}\cdot \text{X5P}-\text{GAP}\cdot \text{F6P}/K_{\text{eqTKL2}}\right)/(\left(K_{1}+\text{E4P}\right)\cdot \text{X}5\text{P}+\left(K_{2}+K_{6}\cdot \text{F}6\text{P}\right)\cdot \text{E4P}+\left(K_{3}+K_{5}\cdot \text{F}6\text{P}\right)\cdot \text{GAP}\\ \;+K_{4}\cdot \text{F6P}+K_{7}\cdot \text{X5P}\cdot \text{GAP}) \end{array}$$

**Table:**

Parameter	Value	Unit	Reference
*K*_1_	0.00184e-3	–	Holzhütter (2004)
*K*_2_	0.3055e-3	mM	Holzhütter (2004)
*K*_3_	0.0548e-3	mM	Holzhütter (2004)
*K*_4_	0.0003e-3	mM	Holzhütter (2004)
*K*_5_	0.0287	–	Holzhütter (2004)
*K*_6_	0.1220	–	Holzhütter (2004)
*K*_7_	0.2150	–	Holzhütter (2004)
*K*_eq_TKL2_	29.7	–	Casazza and Veech (1986)

#### Triose-phosphate isomerase

GAP=DHAP

Flux expression from Marín-Hernández et al. (2011).

$$\begin{array}{ll} V_{r} & =V_{f}/K_{\text{eq}}\cdot K_{\text{mp}}/K_{\text{ms}};\\ {\text{J}}_{\text{TPI}} & =\left(V_{f}\cdot \text{GAP}/K_{\text{ms}}-V_{\text{r}}\cdot \text{DHAP}/K_{\text{mp}}\right)/\left(1+\text{GAP}/K_{\text{ms}}+\text{DHAP}/K_{\text{mp}}\right) \end{array}$$

**Table:**

Parameter	Value	Unit	Reference
*K*_ms_	0.51e-3	M	Marín-Hernández et al. (2011)
*K*_mp_	1.6e-3	M	Marín-Hernández et al. (2011)
*K*_eq_	0.3810	–	Calculated from Marín-Hernández et al. (2011)

### General model parameters

**Table:**

Symbol	Definition	Value	Reference
A	Concentration of ATP + ADP	1.140e-02 M	Marín-Hernández et al. (2011)
CO_2_	Carbon dioxide concentration	2.140e-02 M	Bazil et al. (2010)
GLC_e_	External glucose concentration	1.000e-02 M	Reitzer et al. (1980)
F26P	Fructose-1,6-bisphosphate concentration	4.200e-06 M	Marín-Hernández et al. (2011)
N	Concentration of NAD + NADH	1.345e-03 M	Marín-Hernández et al. (2011)
NP	Concentration of NADP + NADPH	1.932e-05 M	Reitzer et al. (1980)
O_2_	Oxygen concentration	6.500e-05 M	Bazil et al. (2010)
V	Volume	1.000e+05 nl/mg	

### Metabolite concentrations at steady states H and L

**Table:**

Symbol	Model (H)	Experimental (H)	Model (L)
ADP	2.70e-03	2.70e-03^b^	2.83e-03
AMP	3.11e-03	4.00e-03^b^	3.11e-03
ATP	8.70e-03	8.70e-03^b^	8.57e-03
BPG	6.29e-05	9.00e-07^b^	5.41e-05
CIT	1.08e-03	1.70e-03^b^	1.08e-03
DHAP	5.53e-04	0.93e-03^b^	5.53e-04
E4P	9.30e-04	1.60e-05^b^	3.16e-05
F6P	3.62e-05	5.00e-04^b^	4.38e-05
F16P	3.67e-05	3.80e-04^b^	3.68e-04
F26P	2.13e-06	4.20e-06^b^	2.13e-06
G1P	3.41e-03	8.00e-05^b^	3.56e-05
G6P	1.09e-03	1.30e-03^b^	8.08e-04
GAP	1.53e-04	8.00e-05^b^	1.47e-04
GLC	8.97e-04	6.10e-04^b^	7.02e-04
GLY	2.08e-01	1.71e-01^b^	2.08e-01
NAD	1.34e-03	1.34e-03^b^	1.34e-03
NADH	5.00e-06	5.00e-06^b^	5.11e-06
NADP	6.12e-07	0.92e-06^c^	6.14e-07
NADPH	1.87e-05	1.84e-05^c^	1.87e-05
Pi	2.00e-02	4.00e-03^b^	2.00e-02
PEP	5.79e-05	3.20e-04^b^	5.76e-05
PG2	4.98e-06	3.00e-06^b^	5.02e-06
PG3	3.07e-05	6.00e-06^b^	1.83e-05
PGN	1.10e-04	7.50e-05^b^^,^^c^	1.30e-05
PRPP	1.00e-3	1.00e-3^a^	1.00e-3
PYR	1.83e-03	8.50e-03^c^	1.68e-03
LAC	1.55e-02	3.30e-02^a^	1.55e-02
R5P	2.74e-05	1.40e-05^a^	7.47e-06
RU5P	1.43e-04	4.70e-06^a^	1.43e-05
X5P	2.42e-04	1.60e-05^c^	3.50e-05
S7P	8.58e-05	7.00e-05^c^	3.50e-06

*Values are in M unit*.

*^a^Holzhütter (2004)*.

*^b^Marín-Hernández et al. (2011)*.

*^c^Reitzer et al. (1980)*.

### Metabolic fluxes in condition H and L

**Table:**

Reaction	Experimental (H)	Model (H)	Model (L)
AK	NM	0.00	0.00
ATPase	NM	50.6	50.0
DHase	NM	2.65	3.20
DPHase	NM	2.00	1.16
FBA	10.38*	9.24	5.43
ENO	21.24*	18.65	10.90
G6PDH	1.13^a^	1.00	0.58
GAPDH	21.24*	18.65	10.90
GLUT	11.30^a^	10.00	5.98
GP	NM	0.01	0.01
GS	0.11^a^	0.10	0.06
HK	11.30*	10.00	5.98
LDH	18.08^a^	16.00	7.69
MPM	39.5*	33.13	40.0
PFK	10.38*	9.24	5.43
PGDH	1.13*	1.00	0.58
PGI	10.06*	8.91	5.36
PGK	21.24*	18.53	10.90
PGLM	−0.11*	−0.09	−0.05
PGYM	21.24*	18.65	10.90
PK	21.24*	18.65	10.90
PRPPS	0.57^a^	0.50	0.47
R5PI	0.76*	0.67	0.51
RUPE	0.37*	0.33	0.07
TAL	0.16*	0.17	0.04
TKL	0.16*	0.17	0.04
TKL2	0.16*	0.17	0.04
TPI	−10.38*	−9.24	−5.43

*Values are in nmol/min/mg unit. NM, Not measured*.

*^a^Reitzer et al., 1980*.

**Values calculated from the experimental data presented in Reitzer et al. (1980) on the basis of the metabolic network stoichiometry*.

## Research Topic:

From structural to molecular systems biology: experimental and computational approaches to unravel mechanisms of kinase activity regulation in cancer and neurodegeneration

## Topic Editor:

Matteo Barberis, Humboldt University Berlin, Germany; Max Planck Institute for Molecular Genetics, Berlin, Germany
